# Rehabilitation: a key service, yet highly underused, in the management of young patients with sickle cell disease after stroke in DR of Congo

**DOI:** 10.3389/fneur.2023.1104101

**Published:** 2023-05-24

**Authors:** Paul Muteb Boma, Jules Panda, Jean Paul Ngoy Mande, Bruno Bonnechère

**Affiliations:** ^1^Reference Centre for Sickle Cell Disease of Lubumbashi, Institut de Recherche en Science de la Santé, Lubumbashi, Democratic Republic of Congo; ^2^Department of Surgery, Faculty of Medicine, University of Lubumbashi, Lubumbashi, Democratic Republic of Congo; ^3^Department of Neurology and Psychiatry, Faculty of Medicine, University of Lubumbashi, Lubumbashi, Democratic Republic of Congo; ^4^REVAL Rehabilitation Research Center, Faculty of Rehabilitation Sciences, University of Hasselt, Hasselt, Belgium; ^5^Technology-Supported and Data-Driven Rehabilitation, Data Science Institute, University of Hasselt, Hasselt, Belgium

**Keywords:** sickle cell disease, stroke, rehabilitation, management, prevention stroke rehabilitation in sickle cell disease

## Context

Sickle cell disease (SCD) is an inherited disorder that affects red blood cells, causing them to take on a sickle-like shape. This condition can lead to cerebrovascular accidents (CVA) in children, the incidence of which is an estimated 220 to 300 times higher in affected children than in healthy children of the same age. Studies have estimated the prevalence of stroke in this condition to be between 3.7 and 7% (1–3).

Recurrent cerebral infarction can occur in two thirds of SCD patients, usually within 2–3 years after the first stroke (2). Microvascular damage and impaired vasoreactivity can lead to silent infarcts, which are associated with impaired cognitive development (4). A focal neurological deficit that lasts more than 24 h in an SCD child or adolescent is defined as stroke.

It is recommended to screen with transcranial Doppler (TCD) all SCD children from the age of 12-18 months, with further yearly evaluations to identify cerebral vasculopathy before the onset of cerebrovascular accidents (5). Without screening and prophylactic treatment, between 5 and 17% of SCD patients develop a first stroke during childhood or adolescence (6). In 98% of cases, children with SCD survive their first episode of stroke with complete motor recovery, although cognitive sequelae are common. Inadequate management exposes to recurrence of stroke after 12 to 24 months in 67% of patients (3). Chronic transfusions, ideally in partial exchange transfusion mode, reduces this risk to 10% and it has been recently demonstrated that even low-dose hydroxyurea (10 mg/kg/day), cuts the risk by two, demonstrating that these interventions are efficacious for secondary stroke prevention in low-resource settings (7). However, due to its cost and the relatively low acceptance of chronic blood transfusions, these treatments are only used by a minority of patients. A study conducted in Nigeria found that only 10% of caregivers of children who had an indication for chronic blood transfusion for stroke prevention consented to the treatment (8), to the authors' best knowledge there is not such data in the Democratic Republic of Congo (DRC), but according to authors' experience the most important limiting factor is financial.

## Current situation

With an estimated 39,700 SCD births per year, the DRC is the third-most affected country in the world after Nigeria and India (9). The clinical manifestations of SCD are highly variable, as they are influenced by a combination of environmental and genetic factors such as the coexistence of α-triplication and α-homozygous deletion, which leads to less severe forms of SCD (10).

Currently, the only statistics available suggest a prevalence of stroke of 2.7% observed in SCD patients in the south of the country (including Lubumbashi) (11). Despite this, the overall management of SCD remains inadequate, and access to necessary treatments such as hydroxyurea and appropriate blood transfusions is limited due to the precarious socioeconomic status, the very limited availability of hydroxyurea in pharmacies (12), and the scarcity of appropriate blood products (13). Patients with sickle cell disease who have a history of childhood stroke are at an increased risk of global cognitive deficits. Despite the high prevalence of these cognitive disorders documented by several studies, there is no standardized approach to their screening and there is still, currently, a lack of data on rehabilitation for stroke and cognitive difficulties in people with sickle cell disease across the world, including even in high income countries (14, 15).

Therefore, it is crucial to develop effective rehabilitation strategies to minimize the negative impact of stroke on the functions and quality of life of SCD patients. Such strategies should be combined with primary and secondary stroke prevention measures to reduce the overall burden of neurological complications of sickle cell disease, especially in high-prevalence countries such as India and African countries, including DRC (16).

## A lack of resources

Stroke is a major cause of disability that can have a significant impact on people's lives (17, 18). Effective rehabilitation strategies are critical for stroke survivors, as they help to reduce disability, improve quality of life, and support recovery (19). The World Health Organization (WHO) emphasizes the importance of putting health promotion, prevention, and rehabilitation methods in place, as well as strengthening health information systems, evidence, and research (20). Access to high-quality health care, including rehabilitation facilities, has thus been designated as one of the Sustainable Development Goals' pillars (SDGs goal 3) (21). Rehabilitation can be provided in a variety of settings, including inpatient or outpatient hospital settings, private clinics, and community settings such as a person's home. Rehabilitation professionals include physiotherapists, occupational therapists, speech and language therapists and audiologists, orthotists and prosthetists, clinical psychologists, physical medicine and rehabilitation physicians, and rehabilitation nurses, among others (22).

Unfortunately, access to high-quality healthcare and rehabilitation facilities is not always available, particularly in certain parts of the world. For example, in the DRC, there is a ratio of only 1.3 physiotherapists per 100,000 population (23). This means that stroke rehabilitation is often delayed, inadequate, and irregular, resulting in poorer outcomes for stroke survivors. Although it is challenging to create evidence-based guidelines for managing SCD (24), a panel of experts made strong recommendations for chronic complications including use of analgesics and physical therapy for treatment of avascular necrosis (25). Evidence currently suggests the need and positive impact of both motor and cognitive rehabilitation for SCD patients after CVA (26–29). Improving access to healthcare and rehabilitation facilities is therefore essential for ensuring stroke survivors receive the best possible treatment and outcomes. Additionally, healthcare personnel should be educated on the latest (neuro)rehabilitation techniques, so that patients receive the most suitable and effective treatments (30).

The modified Rankin Scale for neurologic disability (mRS) is a tool that has been developed to measures the degree of disability or dependence in the daily activities of people who have suffered a stroke or other causes of neurological disability (31). The mRS is a generic ordinal clinician-rated tool that categorizes the severity and the overall level of disability. This scale rates individuals on 7 levels, ranging from 0 (“no symptom at all”) to 6 (“death”) (32). It is commonly used to describe all dimensions of recovery and disability after acute stroke (33).

## Observations

We conducted a retrospective analysis of the 893 children and adolescents' patients followed at the Sickle Cell Reference Center of Lubumbashi, a medical unit of the Institute of Research in Health Sciences. We collected from the medical records of patients who presented with stroke, the age at the onset of the accident, the sex, the side of hemiplegia, secondary prevention strategy, the delay in rehabilitation after the stroke, the regularity and duration of rehabilitation as well as the mRS assessed 1 year after the stroke during the annual check-up. We performed a linear multivariate regression to try to explain the mRS with the time from stroke to rehabilitation, the duration of the rehabilitation and its frequency adjusted for age.

**Figure 1:**
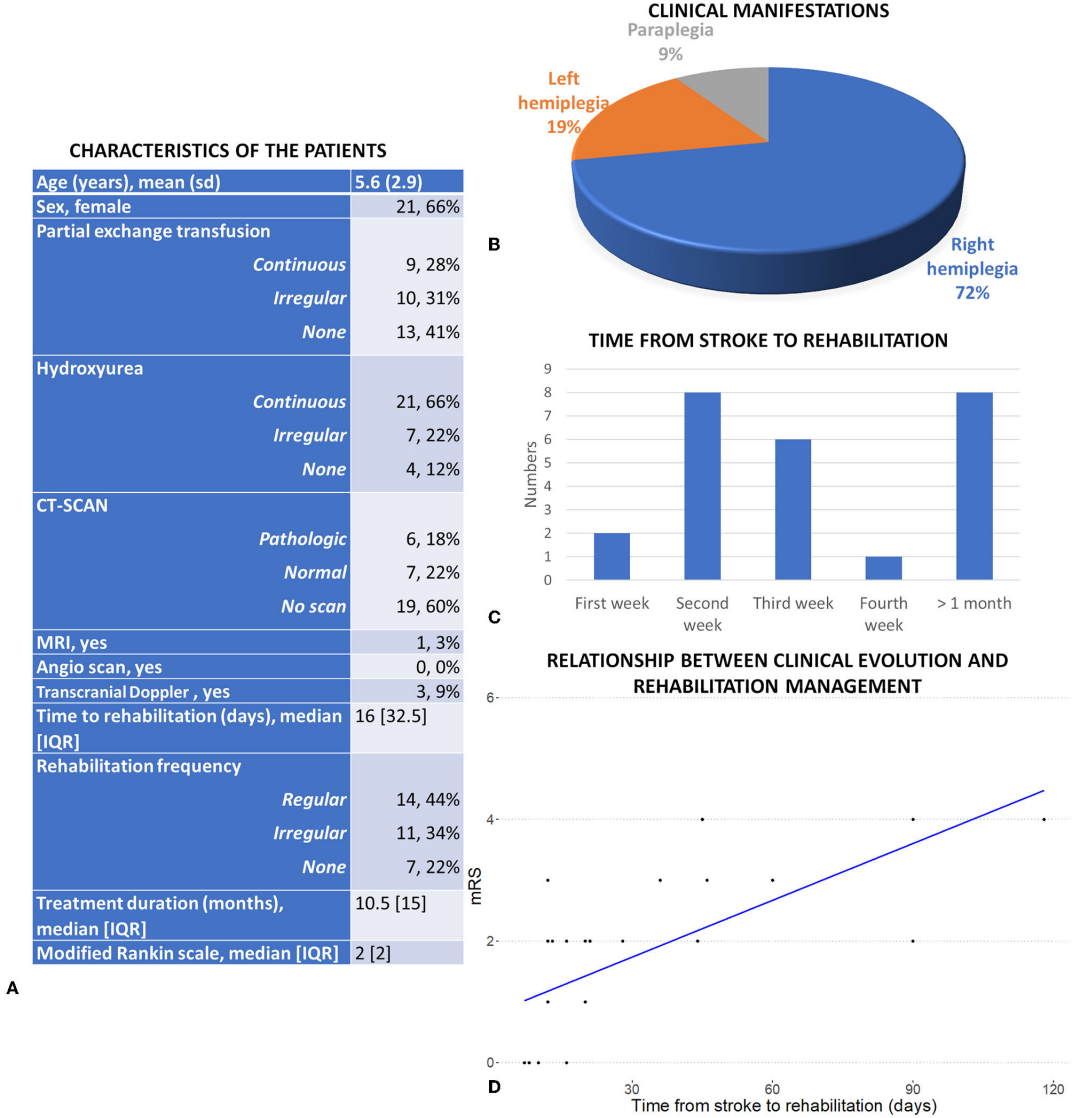
**(A)** Clinical characteristics of the patients and complementary examination. **(B)** Clinical manifestation of the 32 included patients. **(C)** Repartition of the average time from stroke to treatment. **(D)** Relationship between the clinical evolution and the time from stroke to rehabilitation services (after adjustment for frequency, duration of the rehabilitation and age).

A prevalence of 3.6% of stroke (32 out of 890 patients) was observed in our series, with a predominance of male patients (*n* = 21, 66%). These clinical manifestations are illustrated in Table and Figures 1A, B.

Out of the 32 patients who developed a stroke, 25 (78%) have had at least one session of rehabilitation. However, there are two issues concerning the access to these services: most of the patients (14 out of the 25) did not have regularly scheduled sessions, and most of the patients only obtained access to the rehabilitation services long after the stroke (median = 16 days [IQR = 32.5], see Figure 1C). The median duration of rehabilitation was 10.5 months [IQR = 15]. After the initial stroke, 21 out of 32 patients (66%) were regularly treated with hydroxyurea as a secondary preventive measure, which proved to be beneficial. Additionally, among those receiving regular hydroxyurea treatment, only 9 patients (28%) were able to access a long-term transfusion exchange program. The results of the regression show a significant effect of the time from stroke to rehabilitation (ß = 0.028, SE = 0.006, *p* < 0.001) and the frequency (ß = −1.24, SE = 0.36, *p* = 0.002) on the mRS, with a trend for an effect of the duration of the intervention (ß = 0.031, SE = 0.016, *p* = 0.073). These three components explained 73% of the variance in the mRS, highlighting the importance of the rehabilitation service to predict mRS and of an early intervention (Figure 1D).

## Problems to solve

Two major problems stand in the way of improving the rehabilitation of children with SCD suffering from stroke in Lubumbashi. The first point, according to the WHO, relates to the lack of access to rehabilitation services, such as healthcare professionals, infrastructures, and funding (22). The second issue is determining the best rehabilitation strategies adapted to the sequelae present, as the current research in stroke rehabilitation are mainly done with adult populations in high-income countries (34). Addressing these challenges should be an essential part of stroke prevention, which should be established alongside neurorehabilitation programs.

## Potential solutions

Current modalities of rehabilitation include both supervised and unsupervised exercises, but advances in technology are opening new horizons in this field (35, 36). Potential solutions to increase both the quantity and the quality of the rehabilitation include the use of mobile technologies and virtual reality (VR) (37). These solutions broaden the potential of the healthcare sector, at least in high-income nations (38), and can be a low-cost alternative to current rehabilitation modalities, as they do not require immediate access to healthcare clinics or personnel (39). To develop and implement this type of innovative solution, two important points must be addressed: the education and training of healthcare professionals, and the development of local research to develop guidelines adapted to the regional situation (40).

At the WHO level, a rehabilitation competency framework has been developed to provide foundations for curricula for rehabilitation specialists (19). It is important to include new technology tools related to rehabilitation in this framework, as part of the current and future training programs.

In terms of research, it is necessary to look into the acceptance of new technologies as part of medical diagnosis or intervention, as a large portion of the population in low- and middle-income countries still rely on traditional medicine (41). Additionally, raising health literacy levels of patients and the population is important before testing these solutions on a large scale. It is also key to involve the family and community in the management to increase the chances of success, as demonstrated by the community-based rehabilitation approach (42). Regarding the essential preventive method, TCD has emerged as the most effective screening tool for identifying stroke risk in children. This has enabled primary prevention measures, such as blood transfusion and hydroxyurea treatment, to be implemented with success. We believe that hydroxyurea is the most feasible solution in LMICs, especially since its efficacy in other indications shows promising results in improving sickle cell disease morbidity (43). Prescribing it in early childhood can therefore be beneficial for these patients to reduce the risk of stroke.

## Conclusion and call to action

Incorporating VR solutions into rehabilitation services in LMICs is a highly innovative and ambitious project, fraught with potential dangers (44). The challenge is not only to implement VR in rehabilitation, but to raise awareness of the benefits of rehabilitation among healthcare professionals and the population (45). It is clear that technology can play an important role in providing improved healthcare to LMICs. To ensure the successful implementation of (technology-supported) rehabilitation in daily practice, strong local infrastructures need to be developed using North-South consortiums to facilitate inter and multidisciplinary collaborations. Considering the high prevalence of cognitive impairments in both silent cerebral infarcts-associated and non-associated children with SCD, it is reasonable to explore the potential benefits of cognitive rehabilitation interventions for all children with SCD. With the extensive use of mobile phones in the DRC (37), it is possible to conduct high-quality research in this field that could prove cost-effective in the long run without the need for MRI screening. Additionally, this could be a valuable contribution to the field, as a recent systematic review has highlighted the scarcity of research on this topic in developing countries (46). It is interesting to note that randomized controlled trials have conducted on cognitive rehabilitation in children exposed to cerebral malaria (47) and HIV (48), but there is limited data available on SCD. Therefore, it is worth exploring the evidence base for cognitive rehabilitation interventions in children with SCD, with and without cerebral infarction, to potentially benefit children with this condition (49).

In the particular context of SCD, it is important to develop local scientific research capacity and generate evidence supporting the use of rehabilitation, as the vast majority of the patients are in LMICs and therefore there is not enough research performed in HIC due to the lack of patients (and better prevention and control) (50).

Additionally, the development of new affordable and portable technology and solutions can enable continuous patient monitoring and follow-up (51, 52), leading to more individualized rehabilitation (53) and could allow the validation of new interventions or technologies (54). People-centered care is essential for this, as it involves actively involving the patient in the care, providing them with the knowledge and support they need to make decisions and engage in their own care, and ensuring that caregivers are performing optimally within a supportive working environment (55).

To ensure successful integration of these interventions into the healthcare system, a multi-level approach must be taken, with attention to the legal, regulatory, and economic aspects at the macro-level, local health service and community at the meso-level, and the needs of the patient at the micro-level (56). Challenges include providing access to hardware, mobile connection, and education for healthcare practitioners and patients (57), but also the rapid pace of technological change, where hardware and software may quickly become obsolete (58). Additionally, it is essential to consider the financial burden on families who may not be able to afford extended stays in rehabilitation centers. In light of these challenges, it may be worth considering the feasibility of offering rehabilitation services in the home environment. For instance, in the DRC, where mobile phone and tablet use is widespread, utilizing the family's existing technology for cognitive rehabilitation could be a viable option. Such an approach could potentially provide more cost-effective and accessible care to those who require rehabilitation services. However, it is crucial to acknowledge that this approach has its limitations and should be carefully evaluated to ensure that it aligns with the specific needs and context of the population being served.

In regions with a high prevalence of sickle cell disease and stroke, access to rehabilitation for children remains extremely limited. Although the figures presented here are from the southern part of DR Congo, the conclusions can be generalized to other endemic countries like Nigeria and India. It is crucial to prioritize the development of rehabilitation services in these countries for both individual and public health. Furthermore, early detection of cerebral vasculopathy using TCD and MRI allows for the identification of higher-risk patients and implementation of preventive management, such as establishing a chronic transfusion program or prescribing hydroxyurea treatment to minimize the risk of stroke.

VR offers the possibility of functional rehabilitation and stimulation of cognition, while promoting the performance of exercises at home with a better compliance rate. The DRC faces a significant digital divide that exacerbates disparities within and between urban and rural areas, as well as across different provinces (59). Despite this, there has been a noticeable surge in the adoption of affordable mobile phones and digital touch pads connected to the internet in larger cities like Lubumbashi. This trend offers a promising solution for families who cannot afford long-term care in rehabilitation centers, as these devices provide a viable alternative for at-home exercise programs. To improve global health, reduce the burden of CVA, and address the shortage of rehabilitation professionals, new technology must be used. As effective and accessible rehabilitation therapies are lacking in many LMICs, these VR applications could prove to be extremely beneficial in the context of improving the supply of health care, also taking into account access to the necessary disease-modifying treatments, in particular hydroxyurea, which appears to be the easiest to implement.

## Author contributions

PB, JP, and JN carried out the data collection. PB, JP, JN, and BB contributed to the analysis of the results and to the writing of the manuscript. All authors read and approved the final manuscript.
